# Screening for rehabilitation needs in newly diagnosed breast cancer patients: exploring psychometric properties of the National Comprehensive Cancer Network^®^ (NCCN^®^) Distress Thermometer and Problem List

**DOI:** 10.1186/s41687-026-01249-0

**Published:** 2026-11-13

**Authors:** Katarina Velickovic, Pär-Ola Bendahl, Ulrika Olsson Möller, Lisa Rydén, Marlene Malmström

**Affiliations:** 1 Department of Health Sciences, Lund University, Lund, Sweden; 2 Division of Oncology, Department of Clinical Sciences Lund, Lund University, Lund, Sweden; 3 Institute for Palliative Care, Lund University and Region Skåne, Lund, Sweden; 4 Department of Surgery and Gastroenterology, Skåne University Hospital, Lund, Sweden

**Keywords:** Distress Thermometer, Problem List, ROC curve analysis, Psychometrics, Health-related quality of life, Breast cancer

## Abstract

**Background:**

Identifying and managing distress in breast cancer patients is a crucial element of adequate rehabilitation. Immediately after diagnosis, a brief instrument such as the Distress Thermometer has the potential to aid early assessment of distress. Similarly, the accompanying Problem List can aid identification of sources of distress. The aim of this study was to determine cut-off scores of the Distress Thermometer indicating patients with clinically significant impaired functioning which is likely to reflect distress, to investigate its validity, and explore the measurement properties of the Problem List.

**Methodology:**

634 Swedish patients participating in the ReScreen​ intervention study [NCT03434717] were included. ROC curve analysis was performed to explore cut-off scores of the Distress Thermometer in relation to emotional, social, cognitive functioning, and global quality of life subscales of the QLQ-C30. The factor structure of the Problem List was assessed using Confirmatory Factor Analysis. Content validity of the Distress Thermometer was assessed in relation to resilience, assessed using the Connor-Davidson Resilience Scale. Convergent validity of the Problem List was investigated in relation to resilience and relevant QLQ-C30 subscales.

**Results:**

The Distress Thermometer had acceptable accuracy in identifying clinically significant impaired cognitive functioning and global quality of life and excellent relative to emotional and social functioning. A cut-off on the Distress Thermometer between 4 and 5 was found to best balance sensitivity and specificity relative to impaired emotional and social functioning. The Problem List had acceptable model fit. The Distress Thermometer had good content validity and the Problem List convergent validity.

**Conclusions:**

The Distress Thermometer and the Problem List have the potential to aid screening for newly diagnosed breast cancer patients with clinically significant impaired functioning. To ensure the responses accurately reflect the patient’s life, these tools should be used alongside a clinical interview when assessing dysfunction. A cut-off score on the Distress Thermometer between 4 and 5 best balances false negatives and false positives relative to impaired emotional and social functioning. However, before it is implemented in clinical practice, further validation in external and large cohorts is needed. A qualitative evaluation of the instrument would contribute to its utility.

**Supplementary Information:**

The online version contains supplementary material available at https://doi.org/10.1186/s41687-026-01249-0.

## Introduction

Attending to psychological distress is increasingly recognized as one of the necessary elements of care for patients with breast cancer (BC) [1]. Since 1999, NCCN Clinical Practice Guidelines in Oncology (NCCN Guidelines^®^) have advocated for routine screening for distress among cancer patients and introduced screening tools for measuring distress and sources of distress, the Distress Thermometer (DT) and the accompanying Problem List (PL) [1]. NCCN Guidelines^®^ define distress as a complex emotional experience that encompasses a range of psychological (e.g. emotional, cognitive), social, and/or spiritual characteristics that might affect one’s ability to cope with the cancer [1]. Distress exists on a continuum, comprising states from normal feelings of worry and sadness to those that are disabling, such as anxiety, depression, and spiritual crises [1]. A nationwide cohort study in Sweden found that patients with BC are at increased risk of depression, anxiety, and stress-related disorders, especially immediately after diagnosis [2], with an estimated prevalence of depression around 30% [3].

However, it is difficult to establish when normal feelings of sadness and fear become clinically relevant and require referral to a mental health provider and potentially psychosocial support. Given the range of potentially distressing experiences, it is critical to identify patients that experience clinically relevant impaired functioning indicative of distress. Doing so may help identify patients who may not fulfill psychiatric diagnostic criteria, but who are at risk of developing mood disorders. This would help allocate resources to those most in need of rehabilitation. Subsequently, it is important to pinpoint the specific problems experienced by newly diagnosed BC patients.

### The Distress Thermometer

NCCN Guidelines’ DT [1] is a widely used one-item measure which uses a non-pathologizing term (“distress”) and hence places less burden on patients who may feel resistant to extensive questionnaires [4]. Unlike other screening instruments such as PHQ-2 ​[5] and PHQ-9 [6], DT includes a visual analogue scale. The concept of distress also encompasses a broader range of psychosocial problems patients may experience, and not only depressive symptoms. The ease of administration and non-clinical language may thus make DT particularly suitable for the clinical setting. To ensure early detection of patients in need of support, it is crucial to determine whether the DT can identify patients who may meet clinically relevant levels of impaired functioning and require further evaluation in form of a clinical interview. DT was utilized in ReScreen, a complex intervention study designed as an RCT [*NCT03434717*]. The aim of ReScreen is to develop and evaluate a screening-based individualized intervention for highly distressed women with primary BC [7]. The cut-off utilized in ReScreen (between 4 and 5) was based on the distribution of DT scores observed in a small pilot study and hence requires external validation [7].

Several previous studies have examined the accuracy and validity of the DT in multiple cancer populations and different languages [8–11]. The Swedish version of DT has been validated in a broad sample of cancer patients [12] against the Hospital Anxiety and Depression Scale (HADS [13]), used to screen for anxiety and depression in cancer patients. The authors recommend a cut-off score between 3 and 4 [13]. However, newly diagnosed BC patients may experience relatively few issues, and it is therefore important to identify patients that may not yet meet diagnostic criteria for a mood disorder. The EORTC Quality of Life Questionnaire-Core 30 (QLQ-C30 version 3.0 [14]) is a “gold standard” measure that assesses a broad range of issues among cancer populations, commonly used as an outcome measure. Unlike HADS, QLQ-C30 measures a broader spectrum of social, cognitive, and physical problems, which are all experienced during the BC trajectory.

Moreover, it is important to investigate content validity of the DT. One way of assessing content validity of the DT is by investigating its associations with resilience. Resilience is defined as a set of characteristics that allow one to maintain good functioning in face of adversity [15]. Having originated from developmental psychology [16], resilience has thereafter been explored in various populations, including cancer patients [17]. Resilience has been shown to be associated with less emotional distress [18–20] and higher quality of life [19, 20]. Resilience should thus be negatively associated with DT.

### The Problem List

In NCCN Guidelines [1], DT is accompanied by the PL, which aims to identify sources of distress and encompasses different concerns patients may have experienced. It includes practical, family-related, emotional, existential, and physical problem categories, though these vary slightly across different versions of the PL [21]. Sarenmalm and colleagues found that the PL had good model fit and convergent validity in Swedish patients with colorectal cancer [22]. However, unlike colorectal cancer, BC often entails very few physical problems at diagnosis. Before administering the PL in this population, it is important to ensure its validity.

In this paper, we investigate convergent validity of the PL in relation to QLQ-C30 and resilience. Specific problem areas from the PL should have strongest correlations with the corresponding subscales from the QLQ-C30 (i.e., emotional problems with emotional functioning, physical problems with physical functioning, family problems with social and role functioning). Moreover, in previous research, resilience has had a consistently stronger association with mental health-related quality of life outcomes [23] compared to physical health-related outcomes [19, 20]. The number of emotional problems from the PL should thus have a strong association with resilience, whereas other problem areas should have weak-to-moderate associations with resilience.

The aims of this study were to explore the clinically relevant cut-off scores for the DT in relation to emotional, social, cognitive functioning, and global quality of life, as well as investigate the validity of the DT cut-off score utilized in the ReScreen intervention study. Further, the study aimed to explore the content validity of the DT in relation to resilience, factor structure of the PL, and convergent validity of the PL in relation to resilience and HRQoL.

## Methods

### Study participants and procedures

**Fig. 1:**
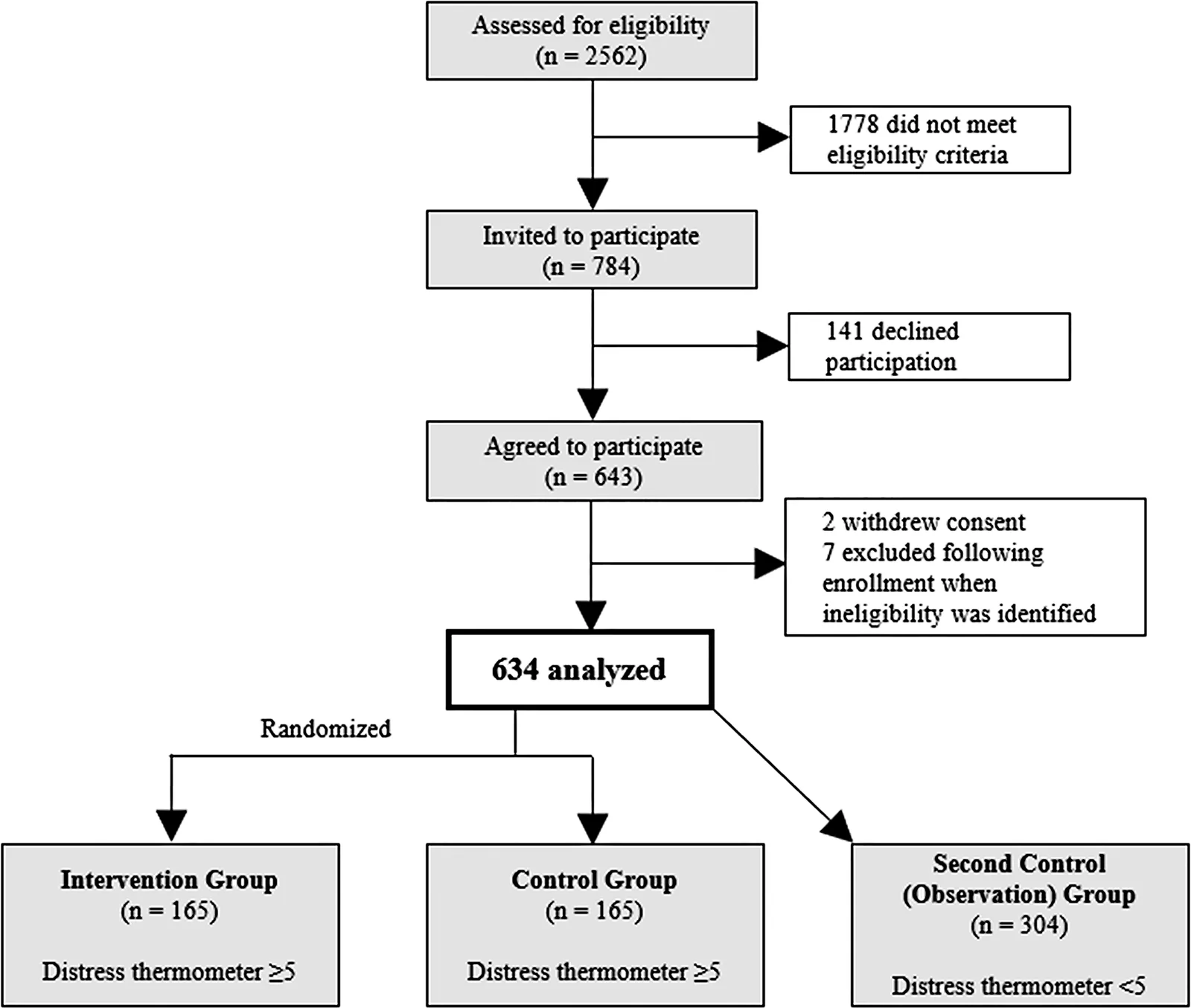
Study flow chart

The present study included 634 newly diagnosed BC patients from the south of Sweden enrolled in the ReScreen​ study​ [7]. The inclusion​ criteria for ReScreen were primary BC diagnosis, planned surgery, age ≥ 18, and ability to communicate in Swedish. The exclusion criteria were pregnancy, recurrent disease, and inability to participate due to cognitive impairment, severe mental illness, or drug addiction [7]. In ReScreen​, patients with high distress were randomized 1:1 between an intervention group and a control group, whereas the patients with low distress constitute a second control group (Fig. 1). The patients randomized to the intervention group followed an individualized evidence-based rehabilitation plan, alongside standard care, whereas the control groups received only standard care. Enrollment lasted from 2019 until 2022. Data collection was conducted at a University hospital and a county hospital, one to two weeks after diagnosis, before treatment. All patients gave written informed consent. Clinical data were extracted from the medical records.

### Measures

#### The NCCN distress thermometer and the problem list [1]

Patients filled in the Swedish version [12] of the NCCN form (Version 2.2013), consisting of DT and PL. The DT [1] measures the level of experienced psychological distress on an 11-point integer scale ranging from 0 (“No distress”) to 10 (“Extreme distress”). As there is no corresponding word in Swedish, the English term “distress” was used, alongside a translated definition supported by a previous study [22]. Patients further filled in the corresponding PL [1], measuring the experience of 39 problems. All problem items are binary, i.e. patients indicate whether they experienced a given problem during the past week. It also contains an open-ended item where patients could report any additional problems.

#### Resilience

Patients filled in the Connor-Davidson Resilience Scale (CD-RISC [15]), a validated widely used 25-item measure of resilience. Respondents indicate whether they agree with a given item on a 5-point Likert integer scale ranging from 0 to 4, 0 representing worse and 4 good functioning. The final score is calculated by summing up all responses, a higher score indicating higher resilience.

#### Health-related quality of life

HRQoL was assessed with QLQ-C30 version 3.0 [14], measuring emotional (EF), physical (PF), role (RF), cognitive (CF), and social functioning (SF), as well as global evaluations of health and quality of life (global QoL) on 4-point and 7-point Likert scales, respectively. It also contains cancer-specific symptom subscales. Final sub-scores are calculated using pre-defined algorithms. For the functional scales, a higher score indicates better functioning, whereas for symptom subscales, a higher score indicates a higher degree of experiencing the symptom.

### Statistical analysis

Means, standard deviations, frequencies, and relative frequencies were calculated for clinical and sociodemographic variables. Distributions of DT scores among patients classified as cases and non-cases using the established QLQ-C30 cut-off scores [24, 25] were graphically presented to aid comprehension of frequencies of patients in each category.

Receiver operating characteristics (ROC) nonparametric curve analysis was performed to investigate accuracy of the DT against QLQ-C30. Subscales of EF, CF, SF, and global QoL were used as criterion variables. The cut-off scores previously demonstrated to represent thresholds for clinical relevance were utilized to identify patients experiencing clinically relevant impaired functioning: EF ≤ 71 [24], CF ≤ 75 [24], SF ≤ 58 [24], global QoL ≤ 80 [25]. The accuracy properties sensitivity, specificity, positive predictive value (PPV), and negative predictive value (NPV) were calculated. Area under the ROC curve (AUC) was also investigated. AUC > 0.70 was considered as acceptable, and > 0.80 as excellent discrimination [26]. An established summary measure which balances sensitivity and specificity, Youden’s J statistic ((sensitivity + specificity) − 1) was calculated to aid identifying the cut-off scores.

Clinical and sociodemographic characteristics were compared between patients below and those above the established cut-off scores using t-tests for independent samples and the Mantel–Haenszel tests of the trend for ordinal variables.

The factor structure of the PL was evaluated using Confirmatory Factor Analysis (CFA), suitable for evaluating an a priori defined latent structure [27–29]. Weighted least square mean- and variance-adjusted estimation method (WLSMV) and tetrachoric correlations were used as they are optimal for binary items [30–32]. Four latent factors were specified, i.e., practical, family-related, emotional, and physical problems. In CFA with binary indicators, squaring standardized factor loadings yields the proportion of variance in the underlying continuous latent response variable explained by the factor, rather than the variance explained in the observed measure [30, 32].

Two items related to fever and substance abuse were excluded as only two participants reported experiencing those problems. The Goodness-of-Fit Indices RMSEA and CFI were utilized when assessing final models, employing criteria of RMSEA < 0.08 [33, 34]; CFI > 0.90 [33].

In the Version 2.2013 of the PL, participants indicate whether they experienced a specific problem in checkboxes labelled “Yes” and “No”. Many participants checked only the “Yes” checkboxes while leaving all “No” checkboxes empty. We assume that missing responses indicated “No” if participants responded “Yes” to at least one of the PL items. Four participants did not respond to any of the PL items. We also ran sensitivity analysis excluding patients who left at least one question unanswered (i.e. neither “Yes” nor “No” box checked) (*n* = 508). An alternative model was subsequently tested, with memory/focus, fatigue, and sleep loading on the emotional instead of the physical factor. The open-ended item for additional problems was not analyzed.

Convergent validity of the PL was investigated using Spearman correlations (*r*) between the total number of experienced problems within each problem category and subscales from QLQ-C30, as well as CD-RISC. Certain items from the PL correspond to symptom subscales from the QLQ-C30. Item-level correlations were performed to check their validity. Additionally, CD-RISC consists of two items that relate to spirituality (item 3 = “Sometimes God or faith can help” and item 9 = “Things happen for a reason”) [15]. Item level correlations between these items and the experience of existential problems were conducted. There were no missing data for the DT. For QLQ-C30, subscores were calculated if a participant filled in at least 50% of the subscale items [14]. Missing scores on QLQ-C30 and PL were handled using listwise deletion (< 1%). Analyses were conducted using Stata 18 [35] and the lavaan package [36] in R version 4.2.2 [37]. Threshold for significance of *p* <.05 [38] was used. The strength of correlations was interpreted as weak (< 0.4), moderate (0.4–0.6) or strong (> 0.6) [39].

## Results

### Frequency and distribution of distress

Sociodemographic and clinical characteristics o​f the sample are portrayed in Table 1.

**Table 1:** Sociodemographic and clinical characteristics of the sample (*N* = 634)

Variable	Number of patients (%)
Type of cancer	
BC in situ	48 (7.6)
Invasive BC	581 (92.4)
Missing values (*n*)	5
Civil status	
Married	362 (57.5)
Cohabiting partner	80 (12.7)
Living apart from partner	27 (4.3)
Single	151 (24)
Other	10 (1.6)
Missing values (*n*)	4
Self-assessed financial situation	
Very bad	8 (1.3)
Bad	23 (3.7)
Neither good nor bad	75 (12)
Good	304 (48.6)
Very good	216 (34.5)
Missing values (*n*)	8
Highest level of education	
Primary school (9 years)	71 (11.3)
Upper secondary school (2 or 3 years)	114 (18.1)
Higher vocational school	117 (18.6)
Undergraduate, basic level	220 (35)
Undergraduate, advanced level	93 (14.8)
Doctoral level	14 (2.2)
Missing values (*n*)	5
Sick leave	
No	493 (79.3)
Yes	48 (7.7)
Not applicable (not eligible for sick leave)	81 (13)
Missing values (*n*)	12
SSRI/Sedative prescription	
No	527 (83.1)
Yes	107 (16.9)
**Variable**	**Mean (SD)**
Age	61.6 (12.1)

Note: SD = Standard deviation

**Fig. 2:**
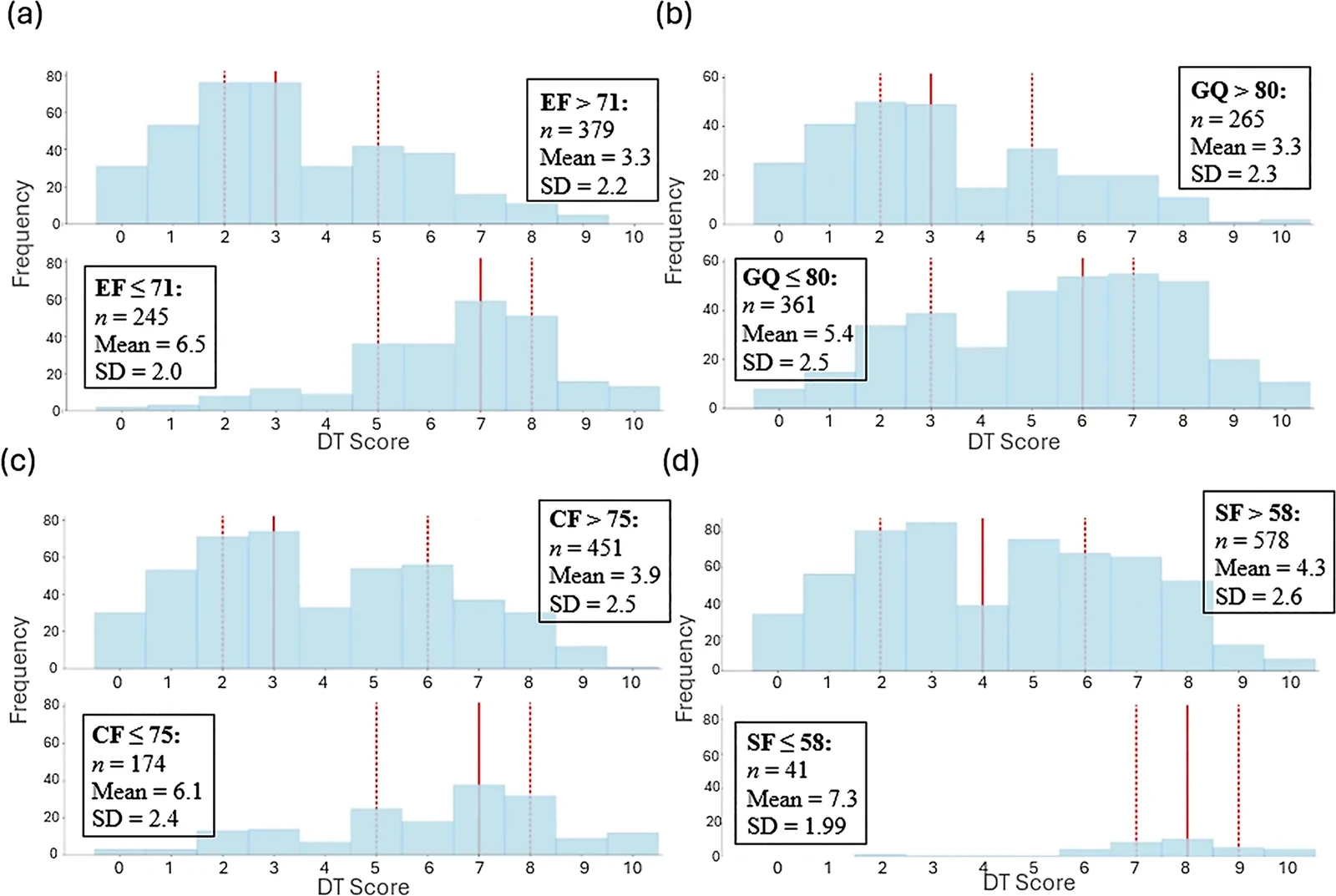
Distribution of the distress thermometer (DT) scores among patients with high and low: emotional functioning (**a**), global QoL (**b**), cognitive functioning (**c**), and social functioning (**d**) subscales from the QLQ-C30 Note: The solid red line represents the median, whereas the dashed red lines represent quartiles

The mean DT score was 4.53 (SD = 2.64). Furthermore, 245 out of 624 (39%) women experienced clinically relevant EF problems (EF < 71), 174 out of 625 (28%) clinically relevant CF problems (CF < 75), 361 out of 626 (58%) had impaired global QoL (global QoL < 80), whilst only 41 out of 619 (7%) experienced clinically relevant SF problems (SF < 58). The distributions of DT scores among patients classified as experiencing clinically significant impaired functioning (cases) and those who were not (non-cases), using the QLQ-C30 subscores, are displayed in Fig. 2.

### Accuracy of the DT

#### Emotional functioning

**Fig. 3:**
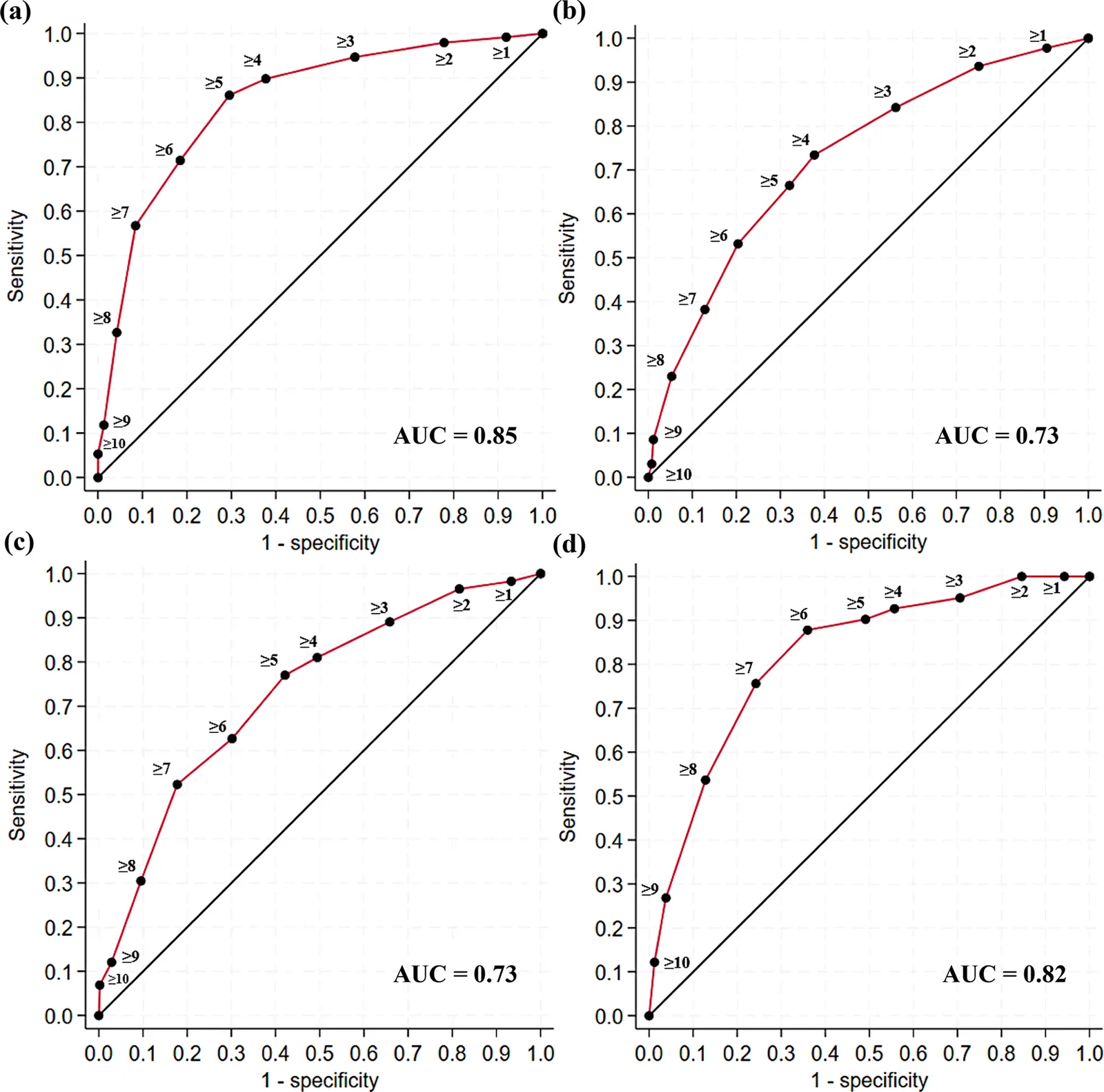
Receiver operating characteristic curves of the Distress Thermometer in relation to emotional functioning (**a**), global QoL (**b**), cognitive functioning (**c**), and social functioning (**d**) subscales from the QLQ-C30

An AUC of 0.85 (Fig. 3, panel a) can be interpreted as 85% probability (95%CI: 0.82–0.88) that a randomly selected patient defined as a case by the EF score will have a higher DT score than a randomly selected patient defined as a non-case, suggesting excellent accuracy of DT for the outcome EF. Youden’s J-index suggested a cut-off between 4 and 5 (non-cases: DT ≤ 4; cases: DT ≥ 5) with sensitivity of 86% and specificity of 70% (Table 2). A total of 330 (52.1%) patients had a score ≥ 5. Using this cut-off, out of those screened as cases by the DT, 35% would be false positives and out of those screened as non-cases, 11% would be false negatives (Table 2).

#### Global QoL

Accuracy of DT against global QoL was acceptable (AUC = 0.73; 95%CI = 0.69–0.77; see Fig. 3, panel b). A cut-off between 3 and 4 (non-cases: DT ≤ 3; cases: DT ≥ 4) was suggested, with sensitivity of 73% and specificity of 62%. A total of 369 (58.2%) patients had a score ≥ 4. Moreover, 27% would be false positives and 37% false negatives (Table 2).

#### Cognitive functioning

Accuracy of DT against CF was acceptable, AUC = 0.73 (95%CI: 0.69–0.77; Fig. 3, panel c). Maximization of Youden’s J-index suggested a cut-off between 4 and 5 for CF (sensitivity = 77%; specificity = 58%; Table 2). This cut-off indicates a large proportion of false positives (58%) whereas 13% would be false negatives (Table 2).

#### Social functioning

AUC = 0.82 (Fig. 3, panel d) which is considered excellent. This analysis suggested a cut-off between 5 and 6 (non-cases: DT ≤ 5; cases: DT ≥ 6) with sensitivity of 88% and specificity of 64%. A total of 252 (39.7%) patients had a score ≥ 6. Using this cut-off indicates a large proportion of false positives (85%), but only 1% of false negatives (Table 2).

**Table 2:** Accuracy properties for the distress thermometer in identifying patients above the previously established cut-off scores on the emotional functioning, global health and QoL, cognitive functioning, and social functioning subscales from the QLQ-C30

Distress Thermometer cut-off	Youden’s J statistic	Sensitivity (%)	Specificity (%)	Correctly classified (%)	PPV (%)	NPV (%)
*Emotional functioning*						
≥ 1	0.07	99.18	8.18	43.91	41.11	93.91
≥ 2	0.20	97.96	22.16	51.92	44.86	94.38
≥ 3	0.37	94.69	42.22	62.82	51.44	92.48
≥ 4	0.52	89.80	62.27	73.08	60.60	90.43
**≥ 5**	**0.57**	**86.12**	**70.45**	**76.60**	**65.32**	**88.70**
≥ 6	0.53	71.43	81.53	77.56	71.43	81.53
≥ 7	0.48	56.73	91.56	77.88	81.29	76.60
≥ 8	0.28	32.65	95.78	70.99	83.33	68.75
≥ 9	0.10	11.84	98.68	64.58	85.29	63.39
≥ 10	0.05	5.31	100	62.82	100	62.03
*Global health and QoL*						
≥ 1	0.07	97.78	9.43	60.38	59.56	75.69
≥ 2	0.18	93.63	24.91	64.54	63.05	75.05
≥ 3	0.28	84.21	43.77	67.09	67.14	67.02
**≥ 4**	**0.36**	**73.41**	**62.26**	**68.69**	**72.63**	**63.19**
≥ 5	0.34	66.48	67.92	67.09	73.87	59.77
≥ 6	0.33	53.19	79.62	64.38	78.07	55.50
≥ 7	0.25	38.23	87.17	58.95	80.25	50.85
≥ 8	0.18	22.99	94.72	53.35	85.59	47.42
≥ 9	0.07	8.59	98.87	46.81	91.20	44.23
≥ 10	0.02	3.05	99.25	43.77	84.73	42.87
*Cognitive functioning*						
≥ 1	0.05	98.28	6.65	32.16	52.85	98.84
≥ 2	0.15	96.55	18.40	40.16	31.28	92.05
≥ 3	0.23	89.08	34.15	49.44	34.40	88.82
≥ 4	0.32	81.03	50.55	59.04	39.13	87.35
**≥ 5**	**0.35**	**77.01**	**57.87**	**63.20**	**41.62**	**86.64**
≥ 6	0.33	62.64	69.84	67.84	44.95	82.95
≥ 7	0.35	52.30	82.26	73.92	52.91	81.46
≥ 8	0.21	30.46	90.47	73.76	53.85	76.78
≥ 9	0.09	12.07	97.12	73.44	60.87	73.92
≥ 10	0.07	6.90	99.78	73.92	100	73.44
*Social functioning*						
≥ 1	0.06	100	5.71	11.95	7.41	100
≥ 2	0.15	100	15.40	21.00	8.13	100
≥ 3	0.24	95.12	29.41	33.76	9.15	98.72
≥ 4	0.37	92.68	44.29	47.50	11.11	98.82
≥ 5	0.41	90.24	50.87	53.47	12.15	98.55
**≥ 6**	**0.52**	**87.80**	**64.01**	**65.59**	**15.54**	**98.61**
≥ 7	0.51	75.61	75.78	75.77	19.25	97.68
≥ 8	0.41	53.66	87.20	84.98	23.82	96.17
≥ 9	0.23	26.83	96.19	91.60	33.69	94.59
≥ 10	0.11	12.20	98.79	93.05	47.46	93.73

Note: Rows in bold represent optimal cut-off scores as measured by Youden’s Index

Patients identified as cases (DT ≥ 5) according to the cut-off between 4 and 5 had worse financial status than non-cases (DT ≤ 4) (*p* <.001). Out of the patients identified as cases, 12.2% were on sick leave (*n* = 39), whereas of the non-cases, 3.0% were on sick leave (*n* = 9) (*p* <.001). Out of cases, 20.3% (*n* = 67) were taking sleeping pills or SSRIs, compared to 13.2% among non-cases (*n* = 40) (*p* <.001). Evidence against the null hypothesis was weaker for the level of education (*p =*.06) and marital status (*p* =.92).

### The Problem List

The most common problems were emotional problems, sleeping problems, and fatigue. The least common problems were fever and substance abuse, hygiene, ability to have children, and childcare. Physical problems were uncommon (Table 3).

All problems were more frequently endorsed by patients defined as cases (DT ≥ 5), except for fever and substance abuse which were only experienced by two patients (Table 3). The difference was strongest for depression, as 91% of patients who experienced depression belonged in the group of patients with DT ≥ 5.

**Table 3:** Frequencies of Problem List items in the total sample (*N* = 634), subsample scoring DT < 5 (*n* = 304) and subsample scoring DT ≥ 5 (*n* = 330), percentages of endorsed problems in each subsample, factor loadings, and standard errors

Problems	*n* (%)	DT < 5	DT ≥ 5	Factor loading	SE
		*n* (%)	*n* (%)		
Practical problems					
1 Childcare	11 (2)	3 (27)	8 (73)	0.22	0.13
2 Housing	17 (3)	3 (18)	14 (82)	0.72	0.12
3 Financial/insurance	50 (8)	9 (18)	41 (82)	0.72	0.07
4 Transportation	30 (5)	9 (30)	21 (70)	0.60	0.09
5 Work/school	78 (12)	15 (19)	63 (81)	0.65	0.07
6 Treatment decision	144 (23)	38 (26)	106 (74)	0.56	0.07
Family-related problems					
7 Relationship with children	49 (8)	13 (27)	36 (73)	0.49	0.08
8 Relationship with partner	47 (7)	13 (28)	34 (72)	0.72	0.08
9 Ability to have children	9 (1)	2 (22)	7 (78)	0.53	0.07
10 Family health issues	106 (17)	30 (28)	76 (72)	0.66	0.14
Emotional problems					
11 Depression	45 (7)	4 (9)	41 (91)	0.73	0.06
12 Fear	250 (39)	52 (21)	198 (79)	0.79	0.04
13 Nervousness	295 (46)	75 (25)	220 (75)	0.70	0.04
14 Sadness	229 (36)	55 (24)	174 (76)	0.76	0.04
15 Worry	455 (72)	155 (34)	300 (66)	0.84	0.04
16 Loss of interest in usual activities	113 (18)	26 (23)	87 (77)	0.72	0.05
Existential problems					
17 Existential/religious questions	74 (12)	12 (16)	62 (84)		
Physical problems					
18 Appearance	40 (6)	4 (10)	36 (90)	0.68	0.06
19 Hygiene/dressing	7 (1)	1 (14)	6 (86)	0.62	0.06
20 Breathing	45 (7)	11 (24)	34 (76)	0.63	0.06
21 Changes in urination	18 (3)	7 (39)	11 (61)	0.42	0.08
22 Constipation	33 (5)	13 (39)	20 (61)	0.44	0.08
23 Diarrhea	47 (7)	7 (15)	40 (85)	0.57	0.07
24 Eating difficulties	34 (5)	5 (15)	29 (85)	0.54	0.07
25 Fatigue	278 (44)	94 (34)	184 (66)	0.74	0.04
26 Feeling swollen	46 (7)	13 (28)	33 (72)	0.71	0.05
27 Fever	2 (0)	1 (50)	1 (50)		
28 Mobility	57 (9)	21 (37)	36 (63)	0.72	0.05
29 Indigestion	28 (4)	6 (21)	22 (79)	0.68	0.07
30 Memory/attention	185 (30)	49 (26)	140 (76)	0.71	0.04
31 Mouth sores	22 (3)	9 (41)	13 (59)	0.15	0.09
32 Nausea	47 (7)	9 (19)	38 (81)	0.55	0.06
33 Dry/congested nose	74 (12)	26 (35)	48 (65)	0.49	0.06
34 Pain	83 (13)	22 (27)	61 (73)	0.71	0.04
35 Sexual problems	32 (5)	9 (28)	23 (72)	0.65	0.07
36 Dry/itchy skin	76 (12)	31 (41)	45 (59)	0.50	0.06
37 Sleep*	278 (44)	82 (29)	196 (71)	0.58	0.04
38 Substance abuse	2 (0)	2 (100)	0 (0)		
39 Tingling in hands/feet	62 (10)	26 (42)	36 (58)	0.52	0.06

Note: *p* values for all factor loadings were < 0.001 apart from childcare (*p* =.10) and mouth sores (*p* =.08)

### Factor structure of the PL

The model had an acceptable fit (χ^2^ (588) = 1241, *p* <.001; RMSEA = 0.04, CFI = 0.92). Two items had low factor loadings, namely mouth sores (0.15) and childcare (0.22), likely due to these problems being endorsed by few patients (Table 3). Correlations between factors were moderate to high, ranging from 0.49 to 0.78.

The model with sleep, fatigue, and memory problems loading on the emotional problems factor had a comparable model fit (χ^2^ (521) = 1213, *p* <.001; RMSEA = 0.05, CFI = 0.92), and comparable factor loadings to the first model. Analysis on the subsample of patients with no missing values (*n* = 508) yielded comparable results (Supplementary Material).

### Convergent validity of the PL

Correlations between PL areas and functional scales from QLQ-C30 are presented in Table 4. The number of practical problems had weak correlations with EF, RF, CF, and SF. The number of family-related problems had weak correlations with all functional scales except for PF. Number of emotional problems had strong correlations with EF and a moderate correlation with CF, as well as weak correlations with RF and SF. The number of physical problems had weak-to-moderate correlations with PF, RF, and SF, and moderate-to-strong correlations with EF and CF. PF was not associated with practical, family, nor emotional problems.

**Table 4:** Spearman correlations between the problem list areas and QLQ-C30 functional scales

	PF	RF	EF	CF	SF
Practical problems	-0.02	-0.32^**^	-0.31^**^	-0.33^**^	-0.28^**^
Family problems	-0.02	-0.12^*^	-0.26^**^	-0.21^**^	-0.22^**^
Emotional problems	-0.04	-0.28^**^	-0.75^**^	-0.47^**^	-0.37^**^
Physical problems	-0.32^**^	-0.46^**^	-0.59^**^	-0.56^**^	-0.43^**^

Note. *< 0.01, **< 0.001; PF = Physical Functioning; RF = Role Functioning; EF = Emotional Functioning; CF = Cognitive Functioning; SF = Social Functioning

All single items correlated significantly with their corresponding QLQ-C30 subscales (all *p*’s < 0.001). Strong correlations were observed for sleeping difficulty (*r* = .74), constipation (*r* = .73), diarrhea (*r* = .73), nausea (*r* = .69), fatigue (*r* = .64), and pain (*r* = .63). Moderate correlations were found for eating difficulty (*r* = .48) and financial problems (*r* = .43), while breathing difficulty had a weak correlation with the dyspnoea subscale (*r* = .31).

Resilience correlated with the DT and all problem areas except family-related problems (*r* = -.02, *p* =.55). The strongest correlations were found with the number of emotional problems (*r* = -.35, *p* < .001), physical problems (*r* = -.31, *p* < .001), and the DT (*r* = -.31, *p* < .001). Correlation with practical problems was weak (*r* = -.12, *p* =.002). The PL’s existential item was associated with item 3 from CD-RISC (*r* = .12, *p* =.006), but the evidence for its association with item 9 was weak (*r* = -.06, *p* =.13).

## Discussion

Identifying problems early in BC is crucial to be able to implement supportive rehabilitation measures [40]. The aim of this paper was to explore the accuracy and validity of the DT and measurement properties of the PL in this population.

We found that the DT had acceptable accuracy in identifying clinically significant impaired CF and global QoL, and excellent relative to EF and SF, implying that the DT has the potential to be used to identify patients with substantial levels of emotional, social, and cognitive problems who should be referred to a more detailed evaluation and thereafter receive rehabilitation in problematic areas. The analysis yielded different cut-off scores for different outcomes. Selecting a cut-off depends on the context and purpose for which the instrument is used. Usually, lower cut-offs result in fewer false negatives, whereas higher cut-offs result in fewer false positives. If the desired treatment or intervention is deemed to entail risks, or the resources are sparse, it would be more beneficial to minimize false positives. However, if it is crucial to provide the treatment or intervention to all patients who need it, it would be beneficial to minimize false negatives.

In this case, the intervention for distress entails psychosocial care, associated with few risks. Sweden has a tax-funded healthcare system, and all BC patients should have access to free rehabilitation services [41]. Still, as much as 40% of are not aware of their right to rehabilitation [40] and distress management is not specified in Swedish National Cancer Rehabilitation guidelines [41]. It is thus unclear to what extent the needed resources are available.

Therefore, we decided to employ Youden’s J-index to estimate cut-off scores as it balances sensitivity and specificity. The results suggested a cut-off between 4 and 5 relative to EF and CF, between 3 and 4 relative to global QoL, and between 5 and 6 for SF. However, the results suggest that many patients would get support despite not exhibiting SF problems, and more than one third of the patients who need support for their QoL would not be identified. This indicates that the DT should be used with caution. The recommended cut-off relative to EF and CF would result in few patients missing out on needed support.

The accuracy of the DT has previously been assessed [42]. Previous evaluations resulted in a recommended cut-off score between 3 and 4 [42, 43]. The difference between our results and previous research could be due to varying criteria in determining the cut-off, as well as the variety of types and stages of cancer. DT was overwhelmingly analyzed relative to HADS8 [8, 10, 13, 44], and sometimes the Brief Symptom Inventory-18 (BSI-18 [45]) [42, 46]. Both scales measure anxiety and depression in cancer patients. To the best of our knowledge, no studies thus far have compared DT against the QLQ-C30. Unlike HADS and BSI-18, QLQ-C30 enables investigation of not only emotional, but also cognitive and social functioning, which are all components of distress [1].

We found that the DT has the potential to be used to guide rehabilitation early in BC care. Nevertheless, it should be noted that, unlike gold-standard screening instruments such as PHQ-9 [6] and GAD [47], the DT, as well as the proposed cut-offs, have not been clinically evaluated and validated by clinicians or patients. Incorporating a qualitative approach would aid interpretability of the acquired cut-off scores. The DT should thus not be utilized in clinical care until a more substantial clinical evaluation of the instrument is conducted.

Overall, the findings support the appropriateness of the cut-off score utilized in the ReScreen study (between 4 and 5), verifying the results of the pilot study [7​]. Patients identified as cases according to this cut-off experienced more problems than those identified as non-cases, were more likely to be on sick leave, take anxiolytic drugs or antidepressive medication, and had worse financial status, further validating this cut-off. Nevertheless, its generalizability to other countries is questionable due to differences in healthcare systems and availability of rehabilitation. Researchers and clinicians should thus consider broader literature on the DT. Longitudinal examinations are needed to determine the appropriateness of this cut-off in later stages of the BC process. Importantly, the DT should be used alongside other methods such as expert review before interventions are implemented.

CFA results suggested that the proposed model had an acceptable model fit, in line with previous research [22]. Nevertheless, physical problems were rarely endorsed. The population of newly diagnosed BC patients is unique as they have not yet experienced treatment side-effects but have recently experienced a highly stressful life event. Unsurprisingly, emotional and existential problems were most common. Physical problems had the strongest correlations with EF and CF. Perhaps this is due to memory/focus, fatigue, and sleep representing problems that are not only physical, but also psychological, and they were the most commonly experienced problems from this category. This finding brings into question the validity of the physical problems from the PL in this population. The model in which memory/focus, sleep, and fatigue were specified to load on the emotional problem factor had a comparable model fit. These items should be interpreted with caution. Moreover, the PL is regularly updated, with the newest version (Version 2.2025) [1] containing six different spiritual and religious concerns alongside other changes. Fatigue, memory, and sleep problems are, however, still defined under the physical problems, deeming the results of this study relevant also for the newest version. However, the new version should be evaluated in the Swedish context.

As expected, resilience had the highest correlation with emotional problems and the DT. However, its comparatively strong correlation with physical problems was not hypothesized and gives further evidence for psychological problems being overrepresented among the physical problems. The results also suggested that those endorsing item 3 from CD-RISC were more likely to experience existential or religious concerns, highlighting the existential element of BC.

One limitation of this study was the cross-sectional sample, which prevented us from investigating long-term validity of the DT and PL. Moreover, we conducted a large number of comparisons, increasing the risk of type I errors. Further, DT cut-off points have not been validated by clinicians and patients, nor have we calculated Minimal Clinically Important Difference and Minimal Detectable Change estimates, necessitating more research on the clinical utility and predictive validity of these scores. However, the strength of this prospective study was a large sample size, as well as exploring the DT in relation to a variety of outcomes, reflecting the multifactorial nature of distress.

## Conclusions

The study suggests that the DT has at least acceptable accuracy relative to EF, CF, SF, and global QoL from QLQ-C30, suggesting that it has the potential to be used alongside a clinical interview to identify patients experiencing emotional, cognitive, and social problems indicative of distress following a BC diagnosis, with a recommended cut-off between 4 and 5. Nevertheless, further validation is needed to determine clinical utility of the DT as a screening instrument which should be used alongside other triage methods like clinical assessment. The PL had an acceptable model fit and convergent validity, but memory/focus, sleep, and fatigue should be examined separately.

## Supplementary Information

Below is the link to the electronic supplementary material.

Supplementary Material 1

## Data Availability

The datasets generated and/or analysed during the current study are not publicly available due privacy restrictions made by the Ethical Review Authority but are available from the corresponding author on reasonable request.

## Abbreviations

AUC: Area under the ROC curve
BC: Breast cancer
BSI-18: Brief Symptom Inventory-18
CD-RISC: Connor-Davidson Resilience Scale
CF: Cognitive functioning
CFA: Confirmatory factor analysis
DT: Distress Thermometer
EF: Emotional functioning
HADS: Hospital Anxiety and Depression Scale
HRQoL: Health-related quality of life
NPV: Negative predictive value
PL: Problem List
PPV: Positive predictive value
QoL: Quality of life
QLQ-C30: Quality of Life Questionnaire-Core 30
ROC: Receiver operating characteristics
SF: Social functioning
