# Supplementary material for: Screening for rehabilitation needs in newly diagnosed breast cancer patients: exploring psychometric properties of the National Comprehensive Cancer Network® (NCCN®) Distress Thermometer and Problem List
Source: J Patient Rep Outcomes. 2026 Nov 13;10:213. doi: 10.1186/s41687-026-01249-0 (PMC13769793; doi:10.1186/s41687-026-01249-0)
Supplement: Supplementary file 1 [file 41687_2026_1249_MOESM1_ESM.docx]

**Supplementary Material**

**CFA of the PL items on the subsample of patients with no missing values**

Supplementary Table 1. Factor loadings and standard errors of the Problem List items (*n* = 508)

| Problems | Factor loading | SE |
| --- | --- | --- |
|  |  |  |
| Practical problems |  |  |
| 1 Childcare | .24 | .13 |
| 2 Housing | .85 | .10 |
| 3 Financial/insurance | .81 | .07 |
| 4 Transportation | .63 | .10 |
| 5 Work/school | .63 | .08 |
| 6 Treatment decision | .56 | .07 |
| Family-related problems |  |  |
| 7 Relationship with children | .52 | .09 |
| 8 Relationship with partner | .73 | .08 |
| 9 Ability to have children | .46 | .08 |
| 10 Family health issues | .59 | .14 |
| Emotional problems |  |  |
| 11 Depression | .76 | .06 |
| 12 Fear | .79 | .04 |
| 13 Nervousness | .70 | .04 |
| 14 Sadness | .80 | .05 |
| 15 Worry | .80 | .04 |
| 16 Loss of interest in usual activities | .76 | .05 |
| Existential problems |  |  |
| 17 Existential/religious questions |  |  |
| Physical problems |  |  |
| 18 Appearance | .68 | .07 |
| 19 Hygiene/dressing | .63 | .07 |
| 20 Breathing | .68 | .07 |
| 21 Changes in urination | .42 | .09 |
| 22 Constipation | .41 | .09 |
| 23 Diarrhea | .56 | .08 |
| 24 Eating difficulties | .53 | .07 |
| 25 Fatigue | .74 | .04 |
| 26 Feeling swollen | .70 | .06 |
| 27 Fever |  |  |
| 28 Mobility | .74 | .05 |
| 29 Indigestion | .65 | .08 |
| 30 Memory/attention | .74 | .04 |
| 31 Mouth sores | .13 | .10 |
| 32 Nausea | .59 | .07 |
| 33 Dry/congested nose | .50 | .06 |
| 34 Pain | .71 | .05 |
| 35 Sexual problems | .71 | .07 |
| 36 Dry/itchy skin | .55 | .06 |
| 37 Sleep | .59 | .05 |
| 38 Substance abuse |  |  |
| 39 Tingling in hands/feet | .53 | .07 |

Model with fatigue, sleep, and memory problems loading on the emotional factor also had an acceptable model fit (Chi^2^ (521) = 1064, *p* < .001; RMSEA = .04, CFI = 0.93, TLI = 0.92) and all items had loadings > .40.
